# Supplementary material for: Controlling protein function by fine-tuning conformational flexibility
Source: eLife. 2020 Jul 22;9:e57180. doi: 10.7554/eLife.57180 (PMC7375816; doi:10.7554/eLife.57180)
Supplement: Supplementary file 1. — (A) The percentage of open and closed populations and their standard deviation. (B) The ATPase stimulation factors and conformational confinement factors. (C) The transition rates and their confidence intervals. [file elife-57180-supp1.docx]

**Supplementary File 1**

This file contains three supplementary tables.

***Supplementary file 1A:*** *Average populations and their standard deviations were obtained by bootstrapping: out of each dataset, random subsets (or samples) containing two thirds of the total number of traces were drawn with replacement. A histogram was compiled for each subset, and fitted with fit function and fixed peak positions from Figure 2-figure supplement 2. Relative populations were obtained for each histogram. This was repeated for 1000 subsets. We report the means of 1000 such populations, and as an uncertainty measure their standard deviations (rounded upwards to 0.5).*

|  | **Open population [%]** | **Closed population [%]** | **Standard deviation [%]** |
| --- | --- | --- | --- |
| **A577I:** | 49 | 51 | 5.5 |
| Reference: | 87 | 13 | 3.5 |
| **Aha1:** | 52 | 48 | 5.0 |
| Reference: | 76 | 24 | 4.0 |
| **crowding:** | 50 | 50 | 10.5 |
| Reference: | 89 | 11 | 4.5 |

***Supplementary file 1B:*** *Estimation of the contribution of conformational confinement to the observed ATPase stimulation under the discussed three conditions. The ATPase stimulation factor comes from Figure 5. The conformational confinement factor is defined as the ratio of the high-FRET population (See Table 1) with versus without stimulation by mutation, Aha1 or macro-molecular crowding, respectively.*

|  | ATPase stimulation factor | Conformational  confinement factor | Confinement-per-stimulation ratio |
| --- | --- | --- | --- |
| A577I | 7 | 3.9 | **0.6** |
| Aha1 | 17 | 2.0 | **0.1** |
| Crowding | 4.4 | 4.5 | **1.0** |

***Supplementary file 1C: Quantitative values of transition rates and their confidence intervals.*** *All values included in Figure 3 are specified in Hz, following the nomenclature of the main text, i.e. rate ‘01’ specifies the transition rate constant from state ‘0’ to state ‘1’. MLE: maximum likelihood estimator of the specified rate constant; +CI / -CI: 95% confidence bound in positive/negative direction, respectively. [*] In the specified one case, -CI is undefined due to asymptotical behavior of the likelihood ratio (i.e. not crossing the chi-squared threshold of 3.81).*

|  | **Rate** | **MLE** | **+CI** | **-CI** |
| --- | --- | --- | --- | --- |
| **A577I:** | 01 | 0,0381 | 0,0101 | 0,0087 |
|  | 03 | 0,0018 | 0,0060 | undef. [*] |
|  | 10 | 0,2404 | 0,0569 | 0,0491 |
|  | 12 | 0,7949 | 0,1182 | 0,1054 |
|  | 21 | 1,0453 | 0,1452 | 0,1311 |
|  | 23 | 0,1933 | 0,0592 | 0,0494 |
|  | 30 | 0,0075 | 0,0063 | 0,0053 |
|  | 32 | 0,0404 | 0,0107 | 0,0093 |
| Reference: | 01 | 0,0160 | 0,0060 | 0,0050 |
|  | 03 | 0,0035 | 0,0028 | 0,0021 |
|  | 10 | 0,1442 | 0,0440 | 0,0366 |
|  | 12 | 0,4147 | 0,0758 | 0,0654 |
|  | 21 | 0,6925 | 0,1049 | 0,0942 |
|  | 23 | 0,0599 | 0,0456 | 0,0325 |
|  | 30 | 0,0176 | 0,0194 | 0,0144 |
|  | 32 | 0,0482 | 0,0273 | 0,0208 |
| **Aha1:** | 01 | 0,0218 | 0,0049 | 0,0044 |
|  | 03 | 0,0023 | 0,0020 | 0,0016 |
|  | 10 | 0,3531 | 0,0585 | 0,0524 |
|  | 12 | 0,9828 | 0,1079 | 0,0987 |
|  | 21 | 1,2728 | 0,1289 | 0,1193 |
|  | 23 | 0,1860 | 0,0492 | 0,0424 |
|  | 30 | 0,0111 | 0,0038 | 0,0034 |
|  | 32 | 0,0277 | 0,0056 | 0,0051 |
| Reference: | 01 | 0,0207 | 0,0052 | 0,0046 |
|  | 03 | 0,0061 | 0,0024 | 0,0020 |
|  | 10 | 0,2497 | 0,0543 | 0,0468 |
|  | 12 | 0,6376 | 0,0942 | 0,0833 |
|  | 21 | 1,8700 | 0,1991 | 0,2000 |
|  | 23 | 0,2172 | 0,0813 | 0,0674 |
|  | 30 | 0,0101 | 0,0063 | 0,0052 |
|  | 32 | 0,0269 | 0,0087 | 0,0074 |
| **crowding:** | 01 | 0,0034 | 0,0026 | 0,0018 |
|  | 10 | 0,0176 | 0,0143 | 0,0092 |
|  | 12 | 0,0961 | 0,0344 | 0,0269 |
|  | 21 | 0,4184 | 0,1071 | 0,0943 |
|  | 23 | 0,0442 | 0,0406 | 0,0251 |
|  | 32 | 0,0014 | 0,0014 | 0,0008 |
| Reference: | 01 | 0,0059 | 0,0014 | 0,0012 |
|  | 10 | 0,0256 | 0,0071 | 0,0060 |
|  | 12 | 0,1036 | 0,0140 | 0,0126 |
|  | 21 | 0,2818 | 0,0320 | 0,0299 |
|  | 23 | 0,0469 | 0,0149 | 0,0124 |
|  | 32 | 0,0244 | 0,0076 | 0,0064 |
